# Induction of MMP-9 release from human dermal fibroblasts by thrombin: involvement of JAK/STAT3 signaling pathway in MMP-9 release

**DOI:** 10.1186/1471-2121-8-14

**Published:** 2007-05-07

**Authors:** Li Wang, Jianmin Luo, Shaoheng He

**Affiliations:** 1 The First Affiliated Hospital, Shantou University Medical College, Shantou 515041, China; 2 Clinical Experiment Center, the First Affiliated Hospital of Nanjing Medical University, Nanjing, Jiangsu 210029, China; 3 The Laboratory for Neural Repair, Shantou University Medical College, Shantou 515041, China

## Abstract

**Background:**

It has been recognized that dermal fibroblasts and matrix metalloproteases (MMP) play crucial roles in wound healing process in skin. Thrombin was found to stimulate IL-8 release from human dermal fibroblasts (HDFs). However, little is known of the effect of thrombin on secretion of MMPs from dermal fibroblasts. In the present study, the influence of thrombin on proMMP-2 and proMMP-9 activity release from primary cultured HDFs, and its potential signaling pathways were investigated.

**Results:**

The results showed that thrombin induced proMMP-9, but not proMMP-2 release from HDFs in a dose dependent manner at 6 h following incubation. Thrombin also upregulated expression of proMMP-9 mRNA in HDFs. Hirudin completely abolished the action of thrombin on HDFs. An agonist peptide of protease-activated receptor-1, SFLLR-NH_2 _stimulated an enhanced release of proMMP-9 from HDFs. AG490, an inhibitor of STAT3 inhibited basal and thrombin-provoked proMMP-9 release and phosphorylation of STAT3. PD98059, an inhibitor of MAPK and LY294002, an inhibitor PI3K failed to significantly inhibit thrombin induced proMMP-9 release.

**Conclusion:**

Thrombin is a potent stimulus of proMMP-9 release from HDFs. Thrombin induced proMMP-9 release is most likely through activation of PAR-1. JAK/STAT3 signaling pathway is involved in proMMP-9 release from HDFs.

## Background

The MMPs, also called matrixin, play a key role in the processes of embryonic development, morphogenesis, reproduction, tissue remodeling and tumor invasion and metastasis [1]. MMP-2 and MMP-9 are geletinases, which degrade type IV collagen, a major constituent of basement membranes, denatured interstitial collagens (gelatins), laminin, elastin, and fibronectin [2]. They are secreted as zymogens with a molecular weight of proMMP-2 being 72 kDa and proMMP-9 being 97 kDa. Dermal fibroblasts produce and organize the extracellular matrix of the dermis. They are able to generate MMPs including MMP-2 and MMP-9, collagen, other extracellular matrix components and cytokines upon activation [3].

In recent years, thrombin has been discovered to play important roles in inflammatory and tissue repair processes by influencing vascular and blood cells including endothelial cells [4], fibroblasts [5], vascular smooth muscle cells [6], T lymphocytes [7], eosinophils [8] and monocytes [9]. As a serine protease, thrombin exerts many of its actions through proteolytic activation of its receptors including protease-activated receptor (PAR)-1, PAR-3 and PAR-4 [10]. These receptors can also be activated without proteolytic cleavage using five to six residue peptides corresponding to the new N termini of the cleaved receptors [11]. PARs are 'single-use' receptors: proteolytic activation is irreversible and the cleaved receptors are degraded in lysosomes. Thus, PARs play important roles in 'emergency situations', such as trauma and inflammation, although their other potential downstream signaling targets have not been fully established [12].

Recently, it was demonstrated that human dermal fibroblasts express PAR-1 and PAR-3, and thrombin is able to stimulate IL-8 release from these fibroblasts predominantly through MAPK/ERK and p38 MAPK signaling pathways [13]. However, little is known of influence of thrombin on MMP-9 and MMP-2 release from fibroblasts. We therefore investigate the action of thrombin on MMP-9 and MMP-2 release from primary HDFs and its potential intracellular signaling pathways in the present study.

## Results

### Expression of PARs in HDFs

**Figure 1:**
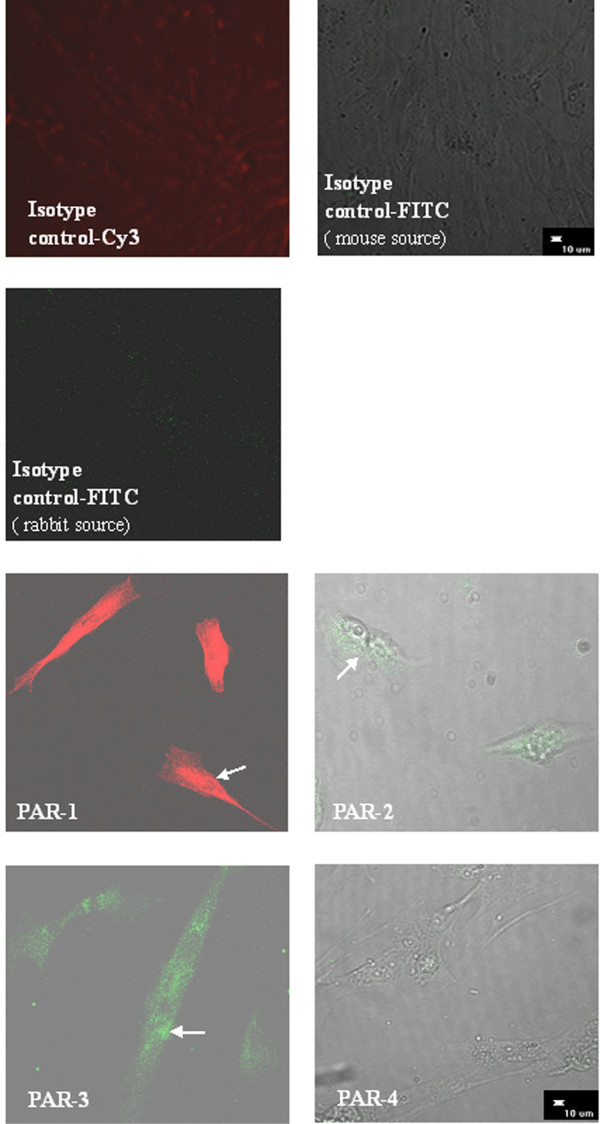
Determination of expression of PAR proteins in HDFs by immunofluorescence staining. HDFs appear to express PAR-1, PAR-2, PAR-3, but not PAR-4 proteins.

**Figure 2:**
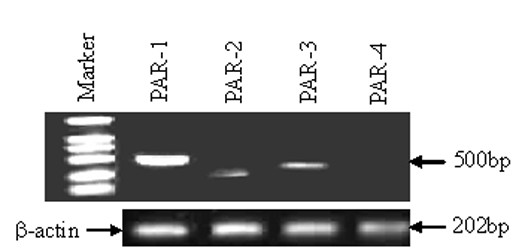
Determination of expression of PAR mRNAs in HDFs by RT-PCR. HDFs express PAR-1, PAR-2, PAR-3, but not PAR-4 mRNAs.

Immunofluorescence staining showed that primary cultured HDFs expressed proteins of PAR-1, PAR-2, PAR-3, but not PAR-4 (Fig. 1). An agarose gel electrophoresis revealed that primary cultured HDFs expressed PAR-1, PAR-2, PAR-3, but not PAR-4 mRNAs (Fig. 2).

### Influence of thrombin on proMMP-2 and proMMP-9 secretion from HDFs

**Figure 3:**
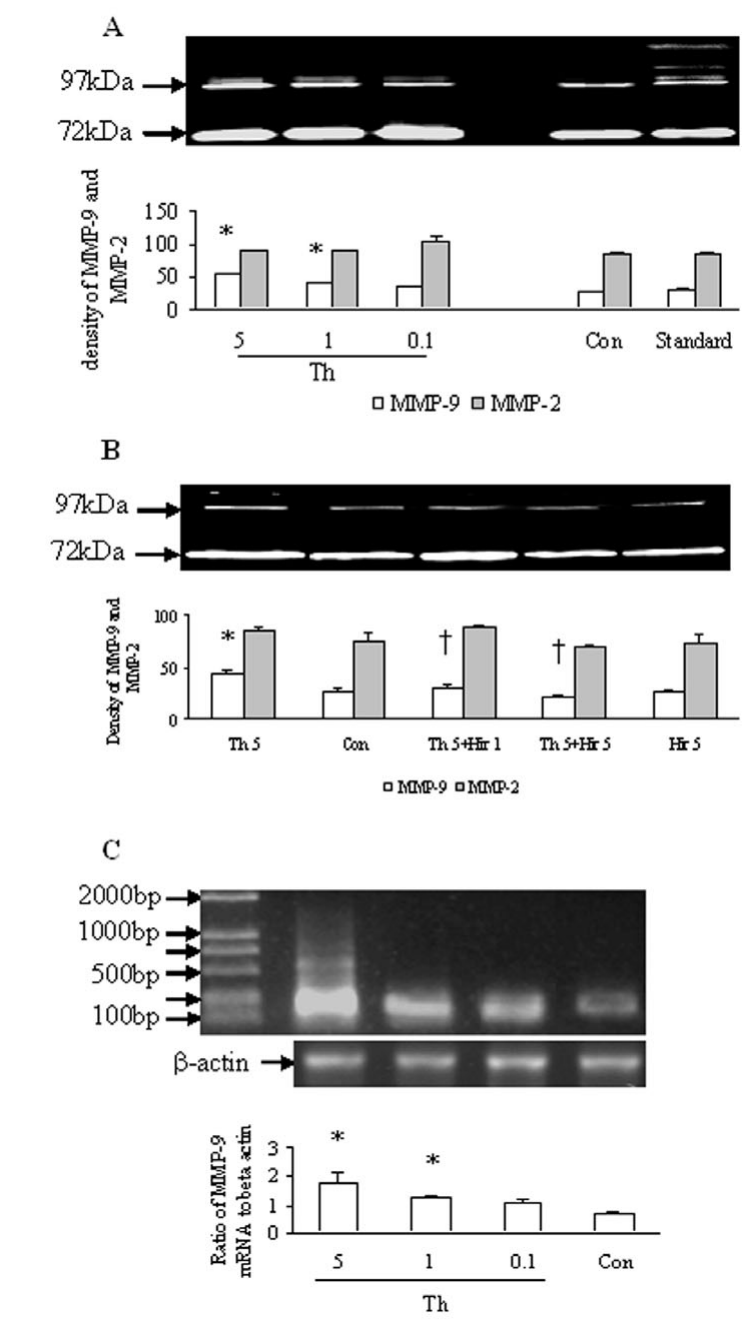
Effects of thrombin (Th, U/ml), hirudin (Hir, U/ml) on release of MMP-2 and MMP-9 activities, and influence of Th on expression of MMP-9 mRNA in HDFs. The cells were incubated with Th, Hir at 37°C for 6 h. Gelatin zymography was used to detect proMMP-2 and proMMP-9 activities. (**A**) MMP-2 and MMP-9 activities released from HDFs in response to various concentrations of Th; (**B**) MMP-2 and MMP-9 activities released from HDFs in response to Th at 5 U/ml and Hir; (**C**) MMP-9 mRNA expression in HDFs in response to various concentrations of Th. The values shown are Mean ± SD for four separate experiments. Cells from each one of the four dermal fibroblast donors were used for one independent experiment. * P < 0.05 in comparison with the response to medium alone (Con); † P < 0.05 in comparison with the response to the corresponding stimulus alone.

Thrombin at the concentrations of 0.1, 1 and 5 U/ml provoked a concentration-dependent increase in proMMP-9 activity from primary cultured HDFs following 6 h incubation. As little as 1 U/ml of thrombin was able to induce significant increase in release of proMMP-9 activity from HDFs (Fig. 3A). The upregulated expression of MMP-9 mRNA in HDFs was found when HDFs were incubated with thrombin at 1 and 5 U/ml for 6 h (Fig. 3C). In contrast, thrombin at the concentrations tested had no siginificant effect either on secretion of proMMP-2 activity (Fig. 3A) or on expression MMP-2 mRNA (data not shown) following 6 h incubation period. Hirudin, a specific thrombin inhibitor at the concentrations of 1 and 5 U/ml completely abolished thrombin-induced release of proMMP-9 activity after 6 h incubation (Fig. 3B).

**Figure 4:**
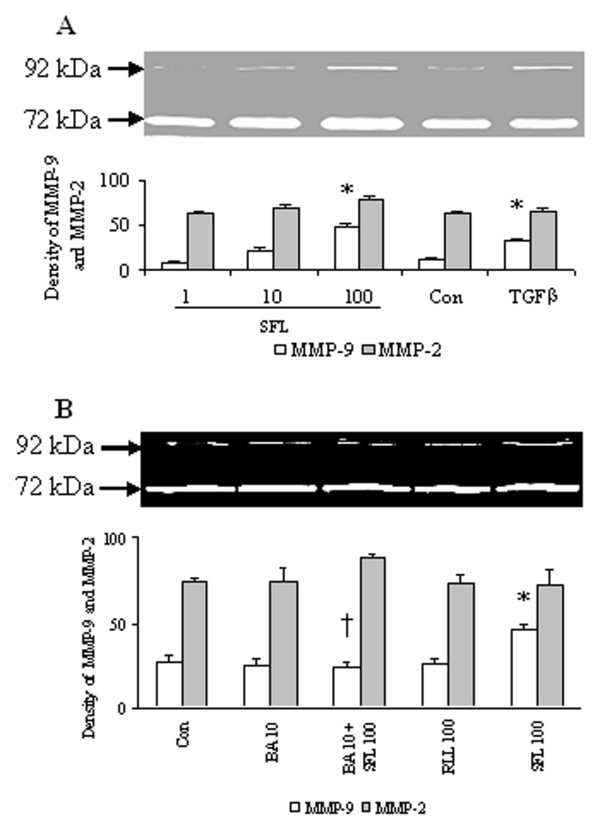
Effects of SFLLR-NH2 (SFL, μM) and RLLFS-NH2 (RLL, μM) on release of MMP-2 and MMP-9 activities in HDFs. The cells were incubated with SFL, RLL or PAR-1 blocking antibody (BA, μg/ml) at 37°C for 6 h, respectively. Gelatin zymography was used to detect proMMP-2 and proMMP-9 activities. (**A**) MMP-2 and MMP-9 activities released from HDFs in response to various concentrations of SFL and TGFβ at 10 ng/ml (as a positive control); (**B**) MMP-2 and MMP-9 activities released from HDFs in response to SFL, RLL and BA. The values shown are Mean ± SD for four separate experiments. Cells from each one of the four dermal fibroblast donors were used for one independent experiment. * P < 0.05 in comparison with the response to medium alone (Con); † P < 0.05 in comparison with the response to the corresponding stimulus alone.

SFLLR-NH_2_, a PAR-1 agonist peptide at the concentration of 100 μM induced a significant release of proMMP-9 activity at 6 h following incubation (Fig. 4A). It appeared that SFLLR-NH_2 _at 100 μM was able to also elicit enhanced secretion of proMMP-2 activity from HDFs though this did not reach statistical significance level (Fig. 4A). As a positive control, TGFβ at 10 ng/ml induced significant release of proMMP-9 activity from HDFs (Fig. 4A). RLLFS-NH_2_, a reverse peptide of SFLLR-NH_2 _had little effect on release of proMMP-9 and proMMP-2 activities from HDFs (Fig. 4B). A PAR-1 blocking antibody completely blocked SFLLR-NH_2 _induced release of proMMP-9 activity (Fig. 4B).

### Effect of PD98059, LY294002 and AG490 on thrombin-induced release of proMMP-9 activity

**Figure 5:**
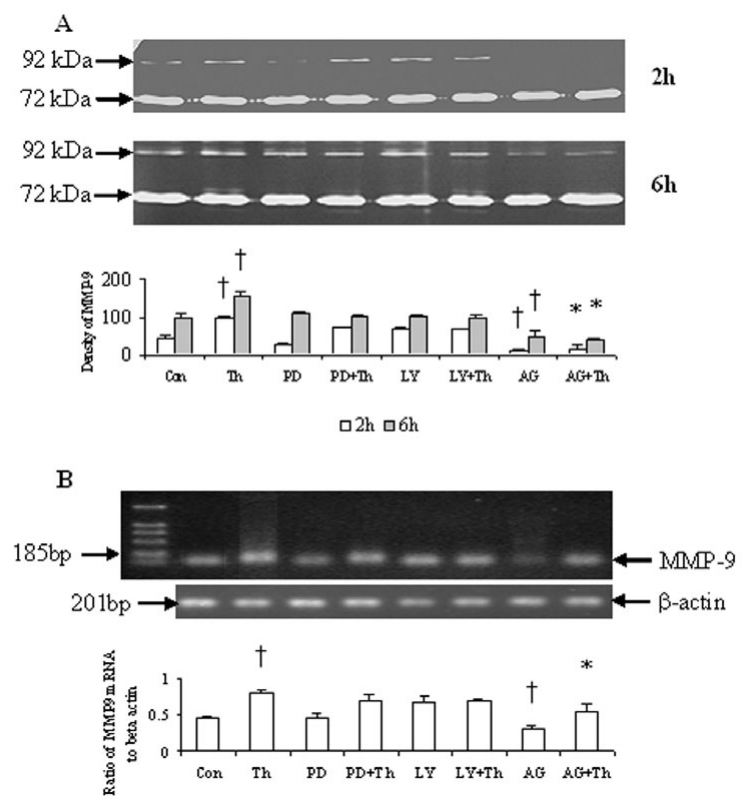
Effect of AG490 (AG), PD98059 (PD) and LY294002 (LY) on MMP-2 and MMP-9 activity release and MMP-9 mRNA expression in HDFs. (**A**) HDFs were incubated with AG490 (40 μM), PD98059 (50 μM) and LY294002 (40 μM) in the presence or absence of thrombin (Th, 5 U/ml) at 37°C for 2 h and 6 h, and MMP-2 and MMP-9 activities were detected with zymograph. (**B**) HDFs were incubated with AG490, PD98059 and LY294002 in the presence or absence of Th at 37°C for 6 h, and MMP-9 mRNA was detected by RT-PCR analysis. The relative levels of MMP-9 mRNA were expressed as the ratio to β-actin. The values shown are Mean ± SD for four separate experiments. Cells from each one of the four dermal fibroblast donors were used for one independent experiment. ^† ^P < 0.05 compared with the response to medium alone (Con); * P < 0.05 compared with the response to thrombin alone.

Following 2 h and 6 h incubation periods, AG490, a JAK/STAT3 pathway inhibitor reduced significantly basal and thrombin-provoked release of proMMP-9 activity (Fig. 5A) and expression of proMMP-9 mRNA in HDFs (Fig. 5B). It was observed also that PD98059, an inhibitor of MAPK pathway and LY294002, an inhibitor of PI3K were able to inhibit thrombin-provoked release of proMMP-9 activity from HDFs though the inhibition does not reach statistical significance level (Fig. 5A). AG490, PD98059 and LY294002 have little influence on basal or thrombin-induced release of proMMP-2 activity from HDFs following both 2 h and 6 h incubation periods (Fig. 5A).

### Effect of AG490 on STAT3 phosphorylation in HDFs

**Figure 6:**
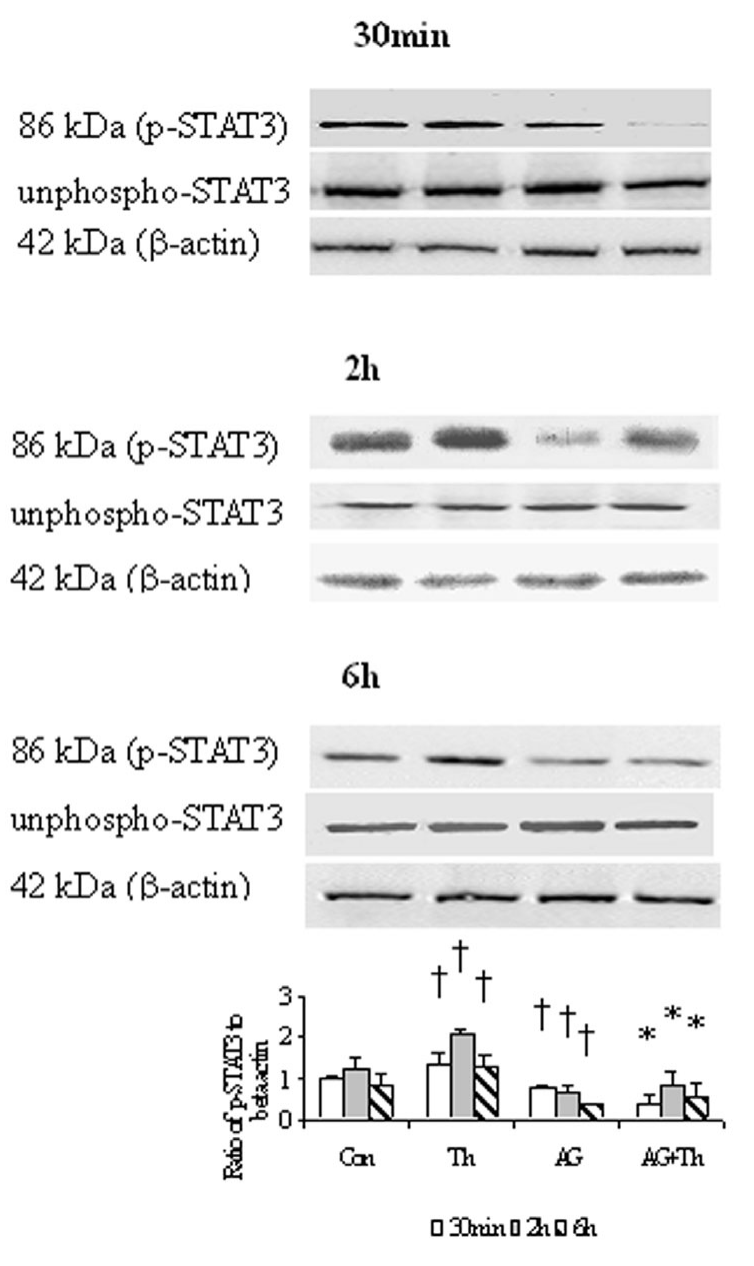
Western blot analysis of effect of AG490 (AG), a JAK/STAT pathway inhibitor on the phosphosrylation of STAT3 in HDFs. Cells were incubated with AG (40 μM), thrombin (Th, 5 U/ml) or Th + AG at 37°C for 30 min, 2 h and 6 h, respectively. Densitometry analysis of immunoblots was carried out using a Scion Image software. The relative levels of phospho-STAT3 were expressed as the ratio to β-actin. The values shown are Mean ± SD for four separate experiments. Cells from each one of the four dermal fibroblast donors were used for one independent experiment. ^† ^P < 0.05 compared with the response to medium alone (Con); * P < 0.05 compared with the response to thrombin alone.

Thrombin at a concentration of 5 U/ml enhanced phosphorylation of STAT3 in HDFs following 30 min, 2 h and 6 h incubation periods. The action of thrombin on JAK/STAT3 pathway was completely abolished by AG490. AG490 also significantly reduced basal phosphorylation of STAT3 in HDFs following 30 min, 2 h and 6 h incubation periods (Fig. 6).

### Effect of PD98059 on ERK 1/2 phosphorylation in HDFs

**Figure 7:**
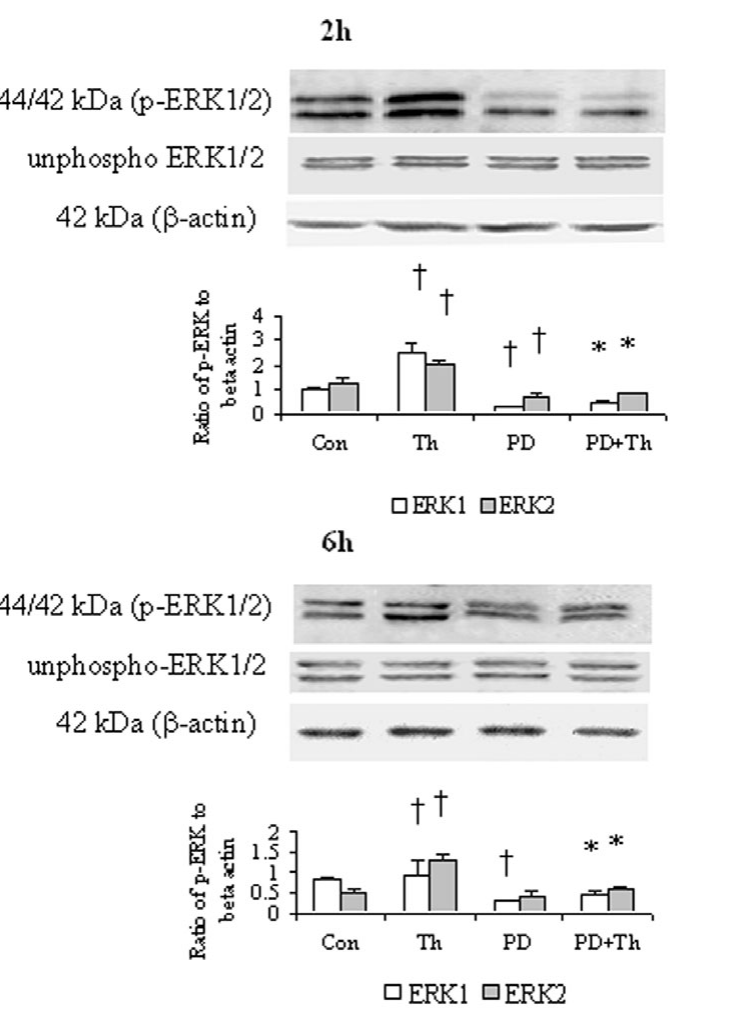
Western blot analysis of effect of PD98059 (PD), a MAPK pathway inhibitor on the phosphorylation of ERK1/2 in HDFs. Cells were incubated with PD (50 μM), thrombin (Th, 5 U/ml) or Th + PD at 37°C for 2 h or 6 h, respectively. Densitometry analysis of immunoblots was carried out using a Scion Image software. The relative levels of phospho-ERK1/2 were expressed as the ratio to β-actin. The values shown are Mean ± SD for four separate experiments. Cells from each one of the four dermal fibroblast donors were used for one independent experiment. ^† ^P < 0.05 compared with the response to medium alone (Con); * P < 0.05 compared with the response to thrombin alone.

Thrombin at a concentration of 5 U/ml induced an enhanced phosphorylation of ERK1/2 in HDFs following 2 h and 6 h incubation periods. PD98059 was able to completely block thrombin induced phosphorylation of ERK1/2 when it was incubated with HDFs for 2 h and 6 h. PD98059 inhibited also basal phosphorylation of ERK1/2 in HDFs following 2 h and 6 h incubation periods (Fig. 7)

### Effect of LY294002 on Akt phosphorylation in HDFs

**Figure 8:**
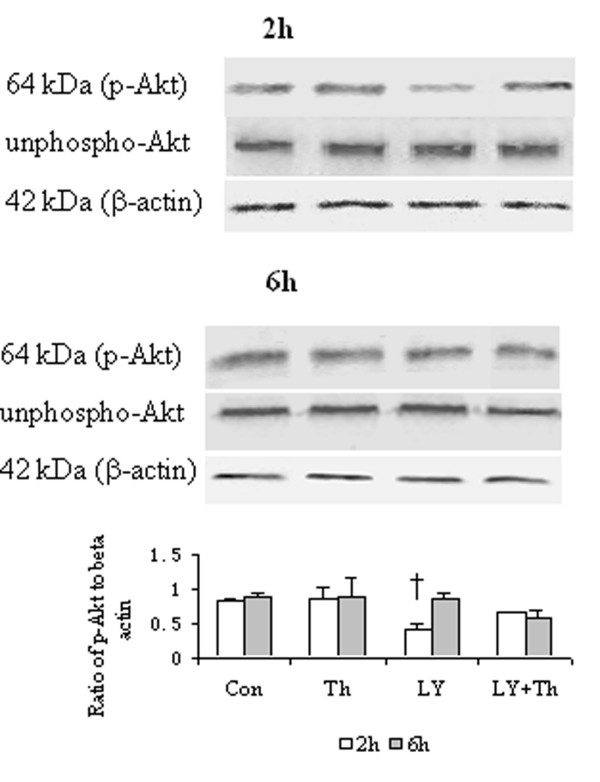
Western blot analysis of effect of LY294002 (LY), a PI3K pathway inhibitor on phosphorylation of Akt in HDFs. Cells were incubated with LY (40 μM), thrombin (Th, 5 U/ml) or Th + LY at 37°C for 2 h or 6 h, respectively. Densitometry analysis of immunoblots was carried out using a Scion Image software. The relative levels of phospho-Akt were expressed as the ratio to β-actin. The values shown are Mean ± SD for four separate experiments. Cells from each one of the four dermal fibroblast donors were used for one independent experiment. ^† ^P < 0.05 compared with the response to medium alone (Con).

Thrombin at a concentration of 5 U/ml failed to induce significantly increased phosphorylation of Akt in HDFs. LY294002 diminished basal phosphorylation of Akt in HDFs at 2 h, but not 6 h following incubation, but had little effect on the action of thrombin in HDFs (Fig. 8).

## Discussion

It was found that thrombin was able to induce proMMP-9 secretion from primary cultured HDFs, indicating that it is likely involved in the tissue remodeling process in the body. In the parallel experiment, thrombin failed to stimulate enhanced proMMP-2 activity release from HDFs, implicating that the action of thrombin on HDFs was a selective one. Selective release of MMP-9 from fibroblasts has been observed previously with rat mast cell sonicate [14], which may support the above view. Since the proMMP-9 activity released from HDFs in response to thrombin at 1 and 5 U/ml and SFLLR-NH_2 _at 100 μM was similar to the activity released in response to TGFβ at 10 ng/ml, it implicated that thrombin was a potent secretogogue of proMMP-9 activity release from HDFs.

It appeared that thrombin induced proMMP-9 release was most likely through activation of PAR-1 as HDFs express PAR-1, PAR-2 and PAR-3, PAR-2 is not a receptor for thrombin and PAR-3 could not be activated by thrombin alone [15]. Our preliminary experiment revealed that a specific agonist peptide of PAR-3, TFRGAP-NH_2 _had little effect on proMMP-9 or proMMP-2 release from HDFs (data not shown). The observations that an agonist peptide of PAR-1, SFLLR-NH_2_, but not its reverse peptide was able to stimulate proMMP-9 release from HDFs, and that PAR-1 blocking antibody completely blocked the action of SFLLR-NH_2 _on HDFs comfirmed that activation of PAR-1 was responsible for thrombin induced release of proMMP-9 from HDFs. The PAR-1 blocking antibody ATAP2 employed in the present study was reported previously as a cleavage-blocking anti-PAR-1 antibody, which abolished completely activated-protein-C induced activation of PAR-1 [16]. It has been reported before that activation of PAR-1 could cause release of IL-8 [13], monocyte chemotactic protein-1 [17] and IL-6 [18] from fibroblasts, which may further support our current finding that stimulation of proMMP-9 release from HDFs by thrombin is likely through activation of PAR-1.

It seems likely that thrombin induced proMMP-9 release was associated with JAK/STAT3, but not MAPK/ERK and PI3K/Akt signaling pathways as inhibitors of STAT3 was able to inhibit thrombin induced proMMP-9 release. The observations that thrombin was capable of stimulating phosphorylation of STAT3, and that AG490 could inhibit thrombin provoked phosphorylation of STAT3 indicate further that thrombin induced proMMP-9 release is associated with JAK/STAT3 signaling pathway in HDFs. However, AG490 itself inhibited basal phosphorylation of STAT3, indicating phosphorylation of STAT3 occurred even when these cells were not under stimulation. Provoking phosphorylation of STAT3 by thrombin has been found with vascular smooth muscle cells previously [19].

Although PD98059, a specific inhibitor of MAPK pathway eliminated thrombin induced phosphorylation of ERK1/2, it failed to significantly inhibit thrombin induced proMMP-9 release from HDFs, indicating MAPK/ERK signaling pathway is unlikely a major pathway for thrombin induced proMMP-9 release. It may serve as a major signaling pathway for thrombin induced other events in HDFs, such as IL-8 release from HDFs[13] and proliferation of astrocytes [20].

## Conclusion

Thrombin is a potent stimulus of proMMP-9 release from HDFs. The action of thrombin on HDFs is most likely through activation of PAR-1. JAK/STAT3 signaling pathway is involved in proMMP-9 release from HDFs. Since MMP-9 presents at elevated levels during early wound healing [21] the finding in the present study may suggest that thrombin is actively involved in tissue remodeling process of new wound in skin.

## Methods

### Materials

Cell culture medium RPMI 1640 and fetal bovine serum (FBS) were obtained from GIBCO BRL (Lifetechnologies, Rockville, MD, USA). PD98059, AG490, LY294002, phospho-p44/42 MAPK antibody, unphospho-p44/42 MAPK antibody, phospho-Akt antibody, unphospho-Akt antibody, phospho-STAT3 antibody and unphospho-STAT3 antibody were purchased from Cell Signaling Technology (Beverly, MA, USA). Human thrombin (T6884), biotin conjugated goat anti-rabbit immunoglobulins, biotin conjugated goat anti-mouse immunoglobulins, extr-Avidin-peroxidase, mouse anti-human β-actin and antibiotics (a mixture of penicillin and streptomycin) were from Sigma (St. Louis, MO, USA). Recombinant hirudin (catalog number 377950) was from Calbiochem (San Diego, CA, USA). PAR-1 agonist peptide (SFLLR-NH_2_) and its reverse peptide (RLLFS-NH_2_) were synthesized at the Xian Meiliang Co. (Xian, China). Mouse anti-human PAR-1, mouse anti-human PAR-2, anti-PAR-3 rabbit polyclonal antibody, anti-PAR-4 rabbit polyclonal antibody, goat anti-mouse IgG-cy3, goat anti-mouse IgG-FITC, goat anti-rabbit IgG-FITC, mouse IgG, rabbit IgG and PAR-1 blocking antibody (ATAP2, against amino acids 42–55 of thrombin receptor of human origin) were from Santa Cruz Biotechnology (Santa Cruz, CA, USA). Sybr green was from Cambrex Bio Science (Baltimore, USA). Most of the other reagents such as salt and buffer components were analytical grade and obtained from Sigma (St. Louis, MO, USA).

### Cell culture

Samples of foreskins were obtained from four healthy donors (aged 18, 18, 20, 21 years, respectively) undergoing circumcision in the First Affiliated Hospital, Shantou University Medical College. The study adhered to the tenets of the Declaration of Helsinki, and all subjects signed an informed consent form before undergoing circumcision. HDFs were isolated by sequential enzymatic digestions and cultured in RPMI 1640 medium supplemented with 10% (vol/vol) FBS, 100 units/ml penicillin, and 100 μg/ml of streptomycin at 37°C in an atomosphere of 5% CO_2 _until confluent islands of cells were observed. Since cells displayed the typical properties of spindle-shaped fibroblasts after the third passage, only passages 3 to 6 HDFs were used for the study. The purity of HDFs was at least 99% as assessed by detecting fibroblast marker using immunostaining with a monoclonal mouse anti-human fibroblast antibody (Dako, Glostrup, Denmark).

### Detection of PARs in HDFs by immunofluorescence staining

Confluent fibroblasts were treated with nonenzymatic cell dissociation fluid to obtain single-cell suspensions. After being seeded onto eight-well chamber slides (Lab-Tek Permanox, Nunc, Life Technologies) at a density of 1 × 10^4 ^cells/well in 300 μl of RPMI 1640 containing 10% FBS, HDFs were incubated in a humidified atmosphere of air containing 5% CO_2 _for 48 h at 37°C, and then fixed with 4% paraformaldehyde for 3 min at room temperature. The fixed cells were incubated with mouse anti-human PAR-1, mouse anti-human PAR-2, rabbit anti-human PAR-3, or rabbit anti-human PAR-4 antibody, respectively for 16 h at 4°C. This was followed by addition of the secondary antibodies including goat anti-mouse IgG-cy3 (for PAR-1), goat anti-mouse-FITC (for PAR-2) or goat anti-rabbit IgG-FITC (for PAR-3 and PAR-4) for 60 mins at room temperature. Mouse IgG or rabbit serum (20 ng/ml) was used as negative controls. After three washes with PBS, the chambers were removed, and coverslips were mounted with an antifade glycerol-PBS-based mountant (Dako, Glostrup, Denmark). The cells were visualized by using a confocal microscope (D-ECLIPSE C1, Nikon).

### Challenge of HDFs with thrombin and SFLLR-NH_2_

The cells were plated in 24-well tissue culture plates (5 × 10^4^/well) and serum starved with serum-free medium overnight. After washing, the fresh medium with various concentrations of thrombin (0.1, 1, 5 U/ml, U = NIH unit, 1 U/ml = 6.7 nM), SFLLR-NH_2 _(1, 10, 100 μM), hirudin (1, 5 U/ml) or thrombin with hirudin was added into each well for 6 h. TGFβ at 10 ng/ml was used as a positive control. The culture supernatant was then collected and stored at -80°C for subsequent analysis.

### RT-PCR analysis of expression of PARs and MMP-9 genes in HDFs

After being exposed to various stimuli for 6 h, the total RNA of HDFs was extracted by using an RNeasy Mini Kit (Qiagen, Germany). cDNA was synthesized from 2 μg of RNA by using ProtoScript First Strand cDNA Synthesis Kit (Biolabs, New England, USA) according to the manufacturer's instructions. The cDNA was amplified using forward and reverse specific primers for amplifying human PARs. β-actin was used as an internal control. Primers were designed based on PAR sequences in Genbank using Omiga software and prepared by BioAsia Co.. The primer sequences were summarized in Table 1. Cycling conditions were as follows: PAR-1, PAR-2, PAR-3, PAR-4, 30 cycles at 94°C for 1 min, 60°C for 1 min, and 72°C for 1.5 min; β-actin was amplified for 30 cycles at 94°C for 1 min, 55°C for 1 min and 72°C for 1 min. The reaction for MMP-9 was allowed to proceed for 30 cycles at 94°C for 30 s, 57°C for 45 s, and 72°C for 45 s. The quality of the PCR product was analyzed on a 1.5% agarose gel, visualized with Sybr green and photographed under UV light. The results were expressed as the ratio of mRNAs of PARs or MMP-9 to β-actin after determination of the density of the bands on agarose gel by densitometer.

**Table 1:** Primer sequences for PARs and MMP-9

Primer		Sequence	Size of product (bp)
PAR-1	sense	5'-CAGCTCCTGGCTGACACTCTTTGTC-3'	500
	antisense	5'-CGAGCAGGGTTTCATTGAGCACAT-3'	
PAR-2	sense	5'-GTGGATGAGTTTCTGCATCTGTCCTCA-3'	328
	antisense	5'-CTGAGGCAGGTCATGGTCATGAAGAGAATGC-3'	
PAR-3	sense	5'-GGCTGGACAGGAGCCACGAT-3'	403
	antisense	5'-AGCGGTTGATGCTGATGCAGG-3'	
PAR-4	sense	5'-GGATCGCCTACCACCTGCGTG-3'	401
	antisense	5'-CCCGTAGCACAGCAGCATGG-3'	
β-actin	sense	5'-AGGGGCCGGACTCGTCATACT-3'	202
	antisense	5'-GGCGGCAACACCATGTACCCT-3'	
MMP-9	sense	5'-CGCCGCTCACCTTCAC-3'	185
	antisense	5'-GCCCAGGGACCACAACT-3'	

### Detection of proMMP-2 and proMMP-9 activities by gelatin zymography

Metalloproteinases generated in culture supernatants were determined using gelatin zymography. Commercial available gelatinases A and B (2 μg; USbiological, New Orleans, LA, USA) were used as standards. Samples or standards were added to platelet-monocyte conditioned medium. The mixture was then placed in 2 × non-reducing SDS sample buffer and electrophoresed in a 10% polyacrylamide gel containing 0.1% gelatin. Following rinsing in 2.5% Triton X-100 for 1 h and in distilled H_2_O for 2 h at room temperature the gel was subsequently incubated at 37°C for 24 h in the substrate buffer containing 50 mmol/L Tris-base (pH 7.6) and 5 mmol/L CaCl_2_. After staining with Coomassie Blue and destaining, the presence of gelatinases in the gel was identified as clear bands on a uniform blue and was quantified by densitometry. The density units were integrated optic density, which was area timed optical density. The ScionImage program was used to quantify the band. Identification of each type of gelatinase on the gel is based on the bands produced by the gelatinase standards.

### Western blot analysis of signal transduction pathways in HDFs upon thrombin stimulation

To determine the optimal concentrations of the inhibitors of signal transduction pathways in HDFs, 25 and 50 μM of PD98059, 20 and 40 μM of LY294002, and 20 and 40 μM of AG490 were preincubated with HDFs for 30 minutes prior to adding 5 U/ml thrombin. Since 50 μM of PD98059, 40 μM of LY294002 and 40 μM of AG490 almost completely abolished thrombin-induced phosphorylation of ERK1/2, Akt and STAT3 respectively (data not shown), whereas 25 μM of PD98059, 20 M of LY294002 and 20 μM of AG490 inhibited thrombin-induced phosphorylation of ERK1/2, Akt and STAT3 by approximately only 30%, 27% and 40% respectively, the higher concentration of each inhibitor was chosen as the optimal concentration throughout the study.

HDFs, which were planted in 12-well tissue culture plates, 1 × 10^6 ^per well, were serum starved overnight and then treated with 50 μM of PD98059, 40 μM of LY294002 or 40 μM of AG490 for 30 min prior to addition of 5 U/ml thrombin. We examined the levels of downstream products of the following pathways: phospho-ERK1/2 in MAPK/ERK pathway, phospho-Akt in the PI3K/Akt pathway, and phospho-STAT3 in the JAK/STAT3 pathway. The levels of these products were determined at 30 min (for phospho-STAT3 only), 2 h and 6 h time points. The levels of proMMP-2 and proMMP-9 in the supernatants were determined by zymography.

The cells were lysed in phosphorylation lysis buffer (0.5%Triton X-100, 150 mM NaCl, 200 μM sodium orthovanadate, 10 mM sodium pyrophosphate, 100 mM sodium fluoride, 1 mM EDTA, 50 mM Hepes, 1.5 mM magnesium chloride, 10% glycerol, 1 mM phenylmethylsulfonyl fluoride, and 10 μg/ml aprotinin) for 60 min at 4°C. After removing cell debris by centrifugation at 10,000 g for 30 min, the supernatants were collected. Equal amount of the cell extracts were loaded on 10% polyacrylamide gel for electrophoresis. Proteins in the gel were then transferred onto polyvinylidene difluoride (PVDF) membranes (Millipore Corporation, Billerica, MA). Following blocking the membranes with 5% skim milk in TBST (50 mM Tris, 0.15 M NaCl, 0.1% Tween 20, pH 7.6) for 1 h at room temperature, the primary antibody was added. Biotinylated goat anti-rabbit immunoglobulins or biotinylated goat anti-mouse immunoglobulins were used as secondary antibodies, which was followed by addition of extr-Avidin-peroxidase. The protein bands were detected by a chemiluminescence method according to manufacturer's instructions (supersignal West Pico, Pierce). Radiographs were photographed with a digital scanning system. The same membranes were stripped at 55°C for 30 min in stripped buffer containing 0.7% β-mercaptoethanol, 2% SDS, and 62.5 mM Tris (pH 6.8) prior to being stained with mouse anti-human β-actin monoclonal antibody as described above.

Densitometry analysis of immunoblots was carried out using ScionImage software. The relative levels of phospho-ERK1/2, Akt, and STAT3 were expressed as the ratio to β-actin.

### Statistical analysis

All data were expressed as mean ± SD. Each experiment was repeated four times. Statistical analysis was performed using one-way ANOVA or Students's *t *test with SPSS (version12.0). *P *value less than 0.05 was considered as statistically significant.

## Abbreviations

Matrix metalloprotease (MMP); Protease-activated receptors (PAR); human dermal fibroblast (HDF); mitogen-activated protein kinase (MAPK); extracellular signal-regulated kinase (ERK); Janus kinase(JAK); signal transducer and activators of transcription (STAT); phosphotidylinositol 3-kinase (PI3K); reverse transcription-polymerase chain reaction (RT-PCR)

## Authors' contributions

LW conceived of and executed the experiments in this study, and drafted the manuscript. JL provided advice with design of experiments and participated in the Westen bolt experiments. SH supervised the study and extensively corrected the manuscript. All authors read and approved the final manuscript.
